# Kinesiology Taping in Grade I–II Meniscus Injuries: A Randomized, Placebo-Controlled Pilot Trial

**DOI:** 10.3390/medicina62010097

**Published:** 2026-01-02

**Authors:** Eren Arabacı, Kübra Okuyucu, Fatih Erbahçeci

**Affiliations:** 1 Physiotherapie Kraft, 45879 Gelsenkirchen, Germany; ernarbc@gmail.com; 2 Graduate School of Health Sciences, Hacettepe University, 06100 Ankara, Türkiye; 3 Department of Physiotherapy and Rehabilitation, Faculty of Health Sciences, Amasya University, 05100 Amasya, Türkiye; 4 Faculty of Physical Therapy and Rehabilitation, Hacettepe University, 06100 Ankara, Türkiye; ferb@hacettepe.edu.tr

**Keywords:** kinesiology taping, pain, quality of life, meniscus injury

## Abstract

*Background and Objectives*: Meniscus injuries, particularly Grade I and II, are common knee injuries that can affect pain, joint function and quality of life, but the effectiveness of non-invasive treatments like Kinesiology taping (KT) in this population remains limited. This pilot randomized controlled trial aimed to explore the short-term effects of KT on pain, fear of movement, muscle strength, proprioceptive force sense, joint range of motion, joint position sense and quality of life in individuals with Grade I/II meniscus injuries. *Materials and Methods*: 26 participants diagnosed with Grade I-II meniscus injury were randomly assigned to two groups: the experimental group was applied ‘Y shaped’ kinesiology taping on quadriceps femoris muscle, based on facilitation technique with 25–50% tension. The control (placebo) group was applied a tape without tension, perpendicular to the quadriceps femoris muscle. Outcomes were evaluated before and 48–72 h after taping. *Results*: Between-group analysis demonstrated a significant improvement in joint position sense at 60° flexion with eyes closed in KT group compared with placebo (*p* = 0.002). Additionally, the KT group showed significantly greater improvements in the physical function (*p* = 0.006) and energy (*p* = 0.013) subdomains of the SF-36 quality of life scale. No significant between-group differences were observed for pain, fear of movement, muscle strength, proprioceptive force sense, or joint range of motion. *Conclusions*: In this pilot study, KT showed acute benefits in proprioception and quality of life in grade I-II meniscus injuries, but no advantage over placebo taping for pain, fear of movement, joint range of motion or muscle strength. Given the exploratory nature and limited sample size, these findings should be interpreted cautiously. Larger trials should confirm these results and determine the role of KT within multimodal rehabilitation programs.

## 1. Introduction

The meniscus, a crescent-shaped fibrocartilaginous structure located in the knee joint, plays a crucial role in load distribution, shock absorption, joint stability, and proprioception [1]. These structures are particularly vulnerable to injury due to their essential biomechanical functions and location within the joint. Meniscus injuries are among the most common knee injuries and can result from acute trauma or degenerative processes [2].

Treatment approaches for meniscus injuries include surgical and conservative methods. Surgical interventions, such as meniscus repair or meniscectomy, are often employed for severe injuries or cases where conservative management fails. However, conservative methods, including manual therapy, exercise, electrotherapy and taping techniques, are the preferred initial approach for Grade I-II meniscus injuries. These less severe injuries typically involve partial tears or minor damage to the meniscal tissue, making them more prone to non-invasive treatments [3,4].

One of the conservative techniques gaining attention in recent years is kinesiology taping (KT). KT is designed to provide mechanical support, enhance proprioceptive input, and potentially reduce pain. Proposed mechanisms include stimulation of cutaneous mechanoreceptors, improved sensorimotor feedback, modulation of the inflammatory process, and alterations in neuromuscular activation patterns [5,6,7]. This method is particularly relevant in meniscus injuries, where proprioceptive deficits and joint instability commonly contribute to functional limitations. Among non-invasive approaches such as exercise therapy, manual therapy, and electrotherapy, KT is often used as an adjunct modality rather than a primary intervention, with mixed evidence regarding its clinical effectiveness [7]. However, the effectiveness of KT remains controversial. Some clinical research suggests potential benefits in postoperative knee recovery and range of motion when KT is used as a lymphatic drainage adjunct [8], whereas other trials in musculoskeletal conditions, such as knee osteoarthritis, have reported minimal or inconsistent effects on pain and function when KT is compared with conventional therapy alone [9].

Despite the growing use of KT in knee osteoarthritis, patellofemoral pain and health populations, evidence regarding its application in meniscus injuries, particularly Grade I–II cases, is limited. Moreover, the acute effects of KT on proprioception, functional outcomes, and patient-reported quality of life in this population remain unclear. Addressing this gap is clinically important, as early improvements in proprioception and function may influence the rehabilitation process [10,11,12].

Therefore, this pilot randomized controlled trial aimed to investigate the short-term effects of KT in patients with Grade I–II meniscus injuries. The primary hypothesis was that KT would lead to greater short-term improvements in pain, knee extension muscle strength, and joint position sense compared with placebo taping. Secondary hypotheses proposed that KT would result in superior improvements in proprioceptive force sense, joint range of motion, fear of movement, and quality of life. These outcome measures were chosen to capture the multidimensional clinical impact of meniscal injuries, reflecting key domains that directly influence knee stability, functional performance, rehabilitation progression, and patient-perceived recovery. By exploring these outcomes, the study aims to provide valuable insights into KT’s potential as a non-invasive intervention into clinical practice.

## 2. Materials and Methods

### 2.1. Study Design and Participants

This randomized controlled pilot trial was designed to compare the acute effects of kinesiology taping (KT) and placebo taping (PT) on functional and quality of life parameters in patients with grade I-II meniscus injuries.

The study population consisted of patients diagnosed with grade I-II meniscus injuries through clinical tests or radiological imaging by a physiatrist/orthopedist at Amasya University Sabuncuoglu Serefeddin Education and Research Hospital.

As this study was designed as a pilot randomized controlled trial, the sample size was determined to provide preliminary estimates of treatment effects and feasibility. A priori power analysis was conducted using GPower 3.1 based on an independent-samples *t*-test, with knee muscle strength selected as the primary outcome [13], assuming a large effect size (Cohen’s d = 0.90), an alpha value of 0.05 and a power of 0.70. This calculation indicated that a minimum of 13 participants per group was required, resulting in a total sample of 26 participants.

Participants diagnosed with grade I-II meniscus injuries who met the inclusion criteria were invited to participate in the study by their physicians. The inclusion criteria included being over 18 years old, having a confirmed diagnosis of grade I or grade II meniscus injury and having no prior surgical intervention on the knee region. Exclusion criteria included the presence of severe visual, hearing, or speech impairments that could hinder participation and a body mass index (BMI) exceeding 35. They were initially informed about the study objectives, procedures, and taping interventions by their treating physicians. Those who agreed to participate were added to a list of volunteer participants after signing the informed consent form. Participants were randomly assigned to groups using a computer-generated simple randomization procedure (www.randomizer.org). Each participant was given a number and entered this program. Then the participants were randomly allocated to one of two groups: the experimental group (KT) or the control group (PT) (Table 1). A simple randomization process was used to prevent selection bias, ensuring that each participant had an equal chance of being assigned to either group. Stratification by age, sex, or affected side was not applied due to the pilot nature and limited sample size of the study.

The study was carried out at Amasya University Sabuncuoğlu Şerefeddin Education and Research Hospital, which was fully equipped to meet the requirements of the research. The trial protocol adhered to the principles outlined in the Declaration of Helsinki. All procedures received approval from the Amasya University Ethics Committee (Approval number: E-76988455-050.01.04-81180 and date: 22 July 2022) and registered with ClinicalTrials.gov, assessed on 3 October 2024 (Registration Number: NCT06637670).

### 2.2. Intervention Procedures

To maintain the integrity of the double-blind design, the researcher conducting assessments (KO) was blinded to group allocation and did not take part in the taping procedures. To maintain assessor blinding despite the visible nature of taping, all evaluations were performed with participants wearing a fully covered taping region, and the assessor was not informed about the taping technique applied. Participants were instructed not to disclose any details regarding the taping during assessment sessions. Blinding was maintained throughout all post-intervention assessments conducted 48–72 h after tape application. All interventions and assessments were performed on the affected side.

The test protocol for each subject included the following steps: pre-test measurements, application of taping for both groups and post-test measurements after 48–72 h of taping. Taping was applied on hairless skin in both groups by a physiotherapist researcher (EA) trained in KT.

Adherence to the taping protocol was monitored throughout the application period (48–72 h). Participants were instructed to keep the tape in place, avoid excessive moisture or friction, and report any discomfort or tape displacement. At the post-intervention assessment, tape adherence and skin condition were visually inspected, and participant self-reports were obtained. No participants withdrew due to discomfort, and no clinically relevant skin irritation was observed.

**Figure 1:**
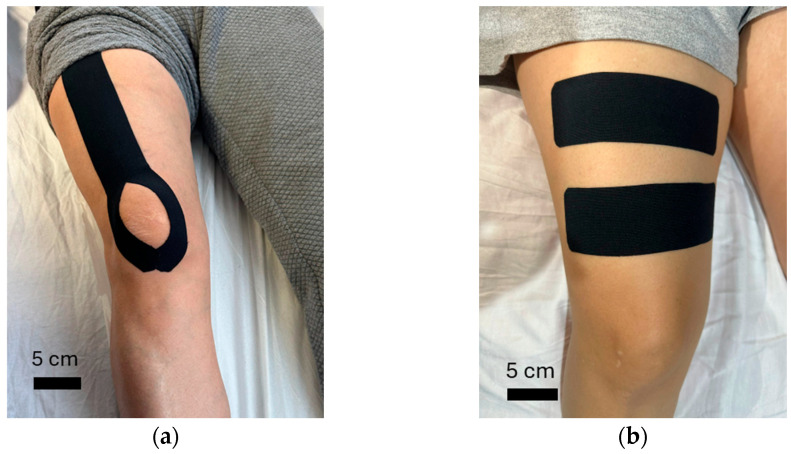
Taping applications: (**a**) Kinesiology taping application; (**b**) Placebo taping application. The scale bar represents 5 cm.

Experimental Group (Kinesiology Taping): Taping was applied to the quadriceps femoris muscle using a “Y-shape” technique. Tape was placed with 25% tension starting from 5 cm below the anterior superior iliac spine, extending over the patella, and finishing below the patella with no tension (Figure 1a). The quadriceps were specifically targeted due to their critical role in dynamic knee stabilization, shock absorption, and control of tibiofemoral loading during functional activities, all of which may be compromised following meniscus injury.

Control Group (Placebo Taping): The placebo taping condition consisted of kinesiology tape applied perpendicular to the quadriceps femoris muscle without tension (Figure 1b). This application was chosen to control for participant expectations and cutaneous sensory stimulation while minimizing biomechanical support, muscle facilitation, or joint stabilization effects, following protocols used in previous KT trials to minimize mechanical and neuromuscular effects [13,14].

### 2.3. Outcome Measures

The primary outcomes of the study are pain, fear of movement and knee extension muscle strength. The secondary outcomes include proprioceptive force sense, joint range of motion, joint position sense, and quality of life. Detailed information on each measurement is given below.

Pain intensity was measured using the Visual Analog Scale (VAS), a validated tool widely employed in clinical research to assess pain [15]. Participants were instructed to mark their perceived pain level on a 10 cm horizontal line, where 0 represented “no pain” and 10 indicated “worst imaginable pain.” Higher scores reflected greater pain severity.

Fear of movement was evaluated using the Tampa Scale of Kinesiophobia (TSK). This scale consists of 17 items scored on a 4-point Likert scale (1 = strongly disagree to 4 = strongly agree). It assesses fear related to movement and injury/re-injury. The total score ranges from 17 to 68, with higher scores indicating greater fear of movement. The Turkish adaptation of the TSK has demonstrated excellent validity and reliability [16].

Knee extension muscle strength was assessed using a handheld dynamometer (MicroFet 2 HHD) in a seated position with knees at 90° flexion. The dynamometer was placed perpendicular to the lower leg, approximately 1–2 cm above the malleolus. Participants were instructed to exert maximum effort for 5 s during each trial, with a 30 s rest interval between attempts. The average of three measurements was recorded in kilograms [17].

Proprioceptive force sense was evaluated using a pressure biofeedback unit (Stabilizer ™, Chattanooga Group Inc., Chattanooga, TN, USA). With the participant in a supine position, the cuff was placed under the knee and inflated to a baseline pressure of 20 mmHg. Participants were first asked to perform a maximal isometric quadriceps contraction, during which the peak pressure value was recorded. 50% of this maximal pressure value was calculated as the target pressure for proprioceptive testing. Participants were then instructed to reproduce the target pressure without visual feedback following a brief familiarization trial. During testing, they were asked to indicate when they believed the target pressure had been reached, at which point the displayed pressure was recorded. The difference between the achieved and target values was recorded in mmHg. Higher deviations indicated poorer proprioceptive accuracy [18].

Knee flexion Range of Motion (ROM) was measured with a goniometer (Baseline^®^, Aurora, IL, USA) while participants were in a prone position. Measurements were taken three times, and the average was recorded in degrees. The goniometer is a standard tool for assessing joint motion, with high inter- and intra-rater reliability [19].

Joint position sense was assessed using a Baseline Digital Inclinometer (Baseline^®^, Aurora, IL, USA). Participants performed knee extension to a target angle of 30° under two conditions: with eyes open and with eyes closed. The repositioning error was calculated as the angular difference between the target and achieved positions. Three trials were averaged for each condition. Greater deviations reflected reduced proprioceptive accuracy. The repositioning error method is widely used in studies of proprioception [20,21].

Quality of life was evaluated using the Short Form-36 Health Survey (SF-36), which measures health across eight domains: physical functioning, bodily pain, role limitations due to physical or emotional problems, vitality, social functioning, mental health, and general health. Domain scores were calculated using raw domain scoring procedures and transformed to a 0–100 scale, with higher scores indicating better perceived health status. Norm-based scoring was not applied in this study. The SF-36 has been extensively validated and adapted for use in Turkish populations [22].

### 2.4. Statistical Analyses

Data were analyzed using IBM SPSS Statistics version 20. Descriptive statistics were presented as means and standard deviations for continuous variables and as frequencies and percentages for categorical variables. The distribution of continuous data was assessed using the Shapiro–Wilk test and visual inspection of histograms. The distribution of all primary and secondary outcome measures met the assumptions of normality (*p* > 0.05), justifying the use of parametric statistical tests.

Between-group comparisons were conducted using change scores (post-intervention minus pre-intervention values) to account for baseline differences between groups. Independent-samples *t*-tests were applied for variables demonstrating normal distribution. Given the exploratory and pilot nature of the study, ANCOVA was not performed, as its assumptions may be unstable in small samples.

No formal adjustment for multiple comparisons was applied, as the primary purpose of this pilot trial was to explore potential intervention effects and estimate effect sizes rather than to perform confirmatory hypothesis testing. Effect sizes were therefore calculated and interpreted alongside *p*-values to support preliminary clinical interpretation according to physiotherapy-specific guidelines, where values of 0.1 indicate a small effect, 0.4 a medium effect, and ≥0.8 a large effect [23,24]. Statistical significance was set at *p* < 0.05, and results were reported with corresponding 95% confidence intervals.

## 3. Results

### 3.1. Participants

**Figure 2:**
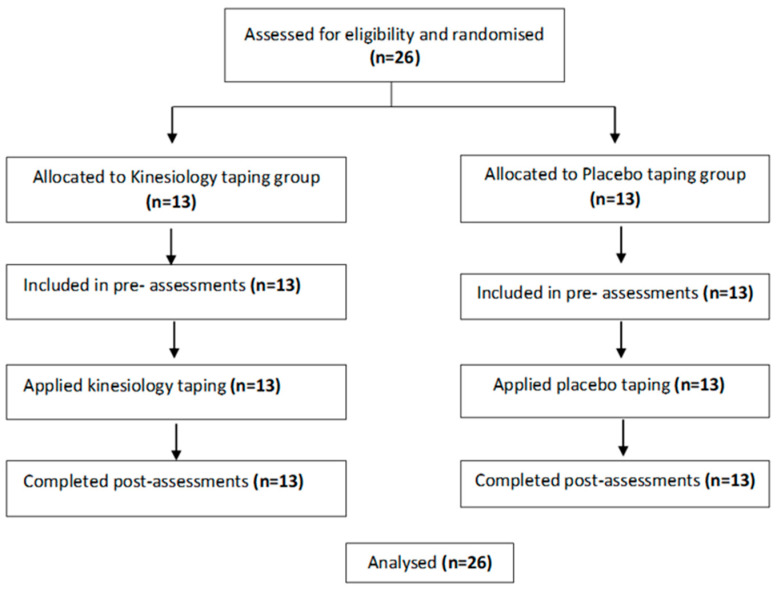
Participant flow diagram.

A total of 26 patients with grade I-II meniscus injuries participated in the study, which took place from August to December 2022. All participants completed the study (Figure 2).

The study included 13 patients in the KT group (mean age ± SD: 41.07 ± 10.82 years) and 13 patients in the PT group (mean age ± SD: 43.92 ± 10.45 years). Table 1 provides an overview of the demographic data for both groups. The physical characteristics of the participants were similar between the KT and PT groups. Data are presented as mean ± standard deviation or as number (percentage).

### 3.2. Measurements

Table 2 displays between-group comparisons of change scores for pain, fear of movement, knee extension muscle strength, proprioceptive force sense, joint range of motion, joint position sense, and quality of life outcomes, along with corresponding effect sizes.

Notably, a statistically significant improvement was observed in joint position sense at 60° flexion with eyes closed in the KT group compared to the PT group (experimental: 3.38 ± 1.44; control: 1.69 ± 1.03; *p* = 0.002), indicating superior performance in the KT group. This difference was associated with a large effect size, indicating a clinically meaningful improvement in proprioceptive accuracy.

Regarding quality of life outcomes, significant between-group differences were detected between the KT and PT groups in two subdomains of the SF-36 scale: physical functioning (experimental: −6.46 ± 3.57; control: −2.92 ± 2.13; *p* = 0.006) and energy levels (experimental: −4.53 ± 2.14; control: −2.61 ± 1.44; *p* = 0.013), both favoring the KT group. These effects were accompanied by medium-to-large effect sizes, suggesting potentially relevant short-term benefits despite the pilot sample size.

No significant between-group differences were found for pain, fear of movement, knee extension muscle strength, or proprioceptive force sense, joint range of motion, or other SF-36 subdomains. Corresponding effect sizes for these outcomes were small, indicating limited or negligible between-group effects over the 48–72 h period.

## 4. Discussion

This study compared the acute effects of kinesiology and placebo taping on various functional and quality of life parameters in patients with grade I-II meniscus injuries. The findings demonstrated that KT was associated with significant improvements in joint position sense at 60° flexion with eyes closed, suggesting enhanced proprioceptive ability in the KT group. Additionally, the KT group showed notable gains in specific subdomains of the SF-36 quality of life scale, including physical functioning and energy levels. However, no significant differences were found between the groups regarding pain, fear of movement, knee extension muscle strength, or proprioceptive force sense, indicating that both interventions had similar effects in these areas.

The findings related to joint position sense at 60° flexion with eyes closed highlight the potential proprioceptive benefits of KT in individuals with grade I-II meniscus injuries by a large effect size, indicating a potentially clinically relevant enhancement in proprioceptive accuracy over a 48–72 h period. KT appears to improve the ability to accurately reposition the knee joint in certain tasks or contexts rather than in general. Clinically, 60° knee flexion corresponds to functional activities such as mid-stance during gait, sit-to-stand transitions, and controlled knee flexion under load. The absence of visual feedback further highlights the potential role of taping in enhancing somatosensory reliance when visual compensation is reduced. This improvement may stem from KT’s capacity to stimulate cutaneous mechanoreceptors and increase somatosensory feedback. However, the finding that KT improved joint position sense only at 60° flexion with eyes closed, but not at 30° flexion or under eyes-open conditions, suggests that its benefits are angle- and condition-specific, making it particularly effective in scenarios requiring a high degree of proprioceptive reliance, such as weight-bearing exercises at specific joint angles. The literature presents mixed findings regarding the acute effects of KT on proprioception. For instance, Wei et al. [25] found no significant impact of KT on knee proprioception in their randomized four-group study involving healthy individuals. Similarly, Torres et al. [21] reported no superior acute effect of KT compared to a control group in healthy participants. Conversely, Cho et al. [26] observed significant acute improvements in proprioception at 15°, 30°, and 45° in patients with knee osteoarthritis, while Ataş et al. [27] also documented enhanced acute proprioceptive outcomes in this patient group. These findings suggest that KT may not significantly improve proprioception in healthy individuals, likely because their baseline proprioception is already optimal. However, its effects appear more pronounced in populations with compromised joint function, such as those with knee deformities [28].

The results showed that KT had potential benefits in patients with meniscus injuries in specific subdomains of the SF-36 quality of life scale, particularly physical functioning and energy levels with medium to large effect sizes, suggesting that KT may positively influence perceived functional capacity and subjective energy levels in the short term. Although these effects were statistically significant, their clinical interpretation must be framed cautiously due to the small sample size and pilot design. Importantly, several outcomes that did not reach statistical significance nonetheless demonstrated small-to-moderate effect sizes, indicating potential trends that may become more apparent in adequately powered trials. The findings still align with existing literature that emphasizes the role of KT in promoting patient-reported outcomes related to physical and mental well-being [29,30]. On the other hand, it is important to note that some studies have reported no significant impact of KT on quality-of-life measures [31,32,33], particularly in populations with severe musculoskeletal dysfunctions and during long-term assessments. This suggests that the benefits of KT may vary based on clinical context, patient characteristics, and whether KT is used as a standalone treatment or as part of a complementary therapy [34]. For instance, KT has demonstrated effectiveness in conditions such as fibromyalgia and chronic venous diseases, underscoring the importance of context in evaluating its efficacy [35,36]. However, these findings should be interpreted with caution. Given the pilot design, limited sample size, and short follow-up period, it is not possible to draw firm conclusions regarding the underlying physiological or psychological mechanisms. The observed changes may reflect short-term perceptual responses, increased body awareness, or contextual effects associated with taping rather than sustained functional adaptations.

The comparative effectiveness of KT versus PT in other outcomes, such as pain, fear of movement, knee strength, and proprioceptive force sense, revealed no significant differences and showed small effect sizes, reinforcing the conclusion that KT does not appear to provide substantial short-term benefits in these domains beyond placebo taping. This finding raises questions about whether the observed improvements in both groups may be explained by non-specific effects of taping, including cutaneous sensory stimulation, increased body awareness, and positive expectancy theory [37,38]. Taping without tension can even provide afferent input, which may influence proprioceptive feedback and perceived function, particularly over short-term exposure periods. This may suggest that the benefits of KT, particularly pain, might be partly influenced by the patient’s belief in treatment. Regarding pain, which is an important clinical outcome, like the findings of this current study, Sedhom [39] and Wageck et al. [31] did not observe any superior effect of KT over PT. The lack of superiority of KT over PT for pain, muscle strength, and fear of movement should be interpreted in light of the pilot design, limited sample size, and short intervention duration, as these outcomes may require longer follow-up periods and greater statistical power to detect clinically meaningful differences [5]. In contrast, studies that have reported significant benefits often conducted measurements after a longer duration, such as one month of KT application [5,32,40]. This suggests that the full advantages of KT may require a longer treatment period to become evident.

This study has several limitations that should be acknowledged. First, a control group that received no intervention was absent. Additionally, it should be acknowledged that placebo taping may still provide some degree of cutaneous sensory stimulation. Therefore, the absence of differences between groups in several outcomes may partly reflect shared sensory or placebo-related effects rather than a lack of intervention efficacy. The findings are specific to the acute effects of KT within a 48–72 h period, and immediate effects or longer-term outcomes were not investigated, which restricts the generalizability of the results to broader timelines. Longitudinal studies assessing the effects of KT over extended periods could provide valuable insights into its role in preventing re-injury and supporting long-term recovery. Another limitation is that all measurements were taken manually, introducing the potential for human error and variability in data collection. Despite these limitations, the study has notable strengths. It was designed as a double-blind, randomized controlled trial to minimize potential biases. The inclusion of multiple assessment parameters, particularly proprioception, was assessed across various joint angles (30° and 60°) and under both open- and closed-eye conditions, providing a comprehensive evaluation of KT’s effects. Additionally, the study uniquely targets patients with grade I-II meniscus injuries, addressing a gap in the existing literature that predominantly focuses on conditions like osteoarthritis, even though meniscus tears are more commonly studied in conjunction with other knee deformities [41,42,43].

### Implications

This pilot study contributes to the growing body of research on KT by exploring its acute effects on grade I-II meniscus injuries, an area that remains underrepresented in sports medicine literature. While KT demonstrated improvements in proprioception at specific angles and quality of life subdomains, its lack of superiority over placebo taping in pain, fear of movement, and strength outcomes aligns with previous findings in osteoarthritis and healthy populations. Accordingly, KT should be considered an adjunctive modality rather than a standalone intervention, particularly in patients who exhibit proprioceptive impairments, sensorimotor control challenges or functional concerns during early phases of conservative rehabilitation, return-to-activity progression, and sports or functional tasks that rely heavily on proprioceptive control. In such contexts, particularly when visual feedback is limited or joint position awareness is compromised, KT may offer short-term sensory facilitation to support neuromuscular rehabilitation. Given the practical ease of KT application, its role in enhancing proprioception and the low risk associated with KT support its selective use alongside exercise-based and multimodal rehabilitation programs, rather than as a primary treatment strategy.

Methodologically, this study provides important information for future trials. The observed effect sizes can inform sample-size calculations, and the feasibility of the 48–72 h taping protocol supports the practicality of short-term application in outpatient and sports rehabilitation settings. Future studies should employ larger, longer follow-up periods and multimodal intervention designs to clarify sustained effects and identify patient characteristics that may predict greater responsiveness to KT.

## 5. Conclusions

This pilot randomized controlled trial demonstrated that KT may provide moderate to large short-term effects on knee joint position sense, particularly at 60° flexion with eyes closed, and certain quality of life subdomains in patients with Grade I and II meniscus injuries. These findings indicate that KT may meaningfully influence proprioceptive acuity and perceived functional capacity in the acute stage. However, outcomes related to pain, fear of movement, muscle strength, and joint range of motion demonstrated small effect sizes and no significant advantage over placebo taping, suggesting limited clinical relevance for these parameters in the short term. Given the small sample size, the pilot nature of the study, and the brief intervention period (48–72 h), the results should be interpreted cautiously. Overall, while KT can be a valuable complementary tool in rehabilitation, its limitations as a standalone intervention underscore the need for comprehensive treatment strategies. Further research is warranted to evaluate its long-term efficacy and optimize its application in diverse clinical settings.

## Figures and Tables

**Table 1:** General characteristics of the participants (*n* = 26).

	Kinesiology Taping Group (*n* = 13)	Placebo Taping Group (*n* = 13)
Age (year)	41.07 ± 10.82	43.92 ± 10.45
Dominant side		
Right	12 (92%)	12 (92%)
Left	1 (8%)	1 (8%)
Injured side		
Right	6 (46%)	6 (46%)
Left	7 (54%)	7 (54%)
Gender		
Female	6 (46%)	6 (46%)
Male	7 (54%)	7 (54%)

**Table 2:** Comparison of Kinesiology and Placebo taping in proprioception, muscle strength, Y balance, horizontal jump, and functional movement.

Assessments	Kinesiology Taping Group (*n* = 13)			Placebo Taping Group (*n* = 13)			*p*-Value †	Cohen’s d
	Pre	Post	Change (Post-Pre, Mean ± SD)	Pre	Post	Change (Post-Pre, Mean ± SD)		
Pain	5.15 ± 1.67	3.38 ± 1.32	−1.76 ± 1.36	4.53 ± 1.56	2.23 ± 1.42	−2.3 ± 1.18	0.29	**0.42**
Fear of movement	39.69 ± 5.67	33.46 ± 9.04	−6.23 ± 5.79	35.92 ± 5.4	33.15 ± 5.09	−2.76 ± 1.42	0.056	**0.82**
Knee extension muscle strength	13.3 ± 2.22	14.86 ± 2.69	1.55 ± 1.44	12.43 ± 1.38	13.3 ± 1.78	0.86 ± 1.01	0.174	**0.55**
Proprioceptive force sense	6.07 ± 2.28	3.38 ± 2.02	−2.69 ± 1.18	4.23 ± 1.48	2.00 ± 1.41	−2.23 ± 1.09	0.31	0.40
Joint range of motion	91.38 ± 5.40	98.46 ± 6.78	7.07 ± 4.68	90.53 ± 5.5	96.3 ± 7.56	5.76 ± 4.32	0.46	0.29
Joint position sense								
30° flexion_Eyes open	5.31 ± 2.56	2.61 ± 1.85	−2.69 ± 1.93	5.54 ± 1.5	2.84 ± 1.72	−2.69 ± 1.6	1	0
30° flexion_ Eyes closed	5.15 ± 1.51	3 ± 1.29	−2.15 ± 1.21	4.76 ± 1.96	2.46 ± 1.89	−2.3 ± 1.31	0.75	0.12
60° flexion_Eyes open	4.38 ± 2.21	2.46 ± 1.5	−1.92 ± 1.32	4.53 ± 2.1	2.46 ± 1.33	−2.07 ± 1.18	0.75	0.12
60° flexion_Eyes closed	5.38 ± 1.55	2 ± 1	−3.38 ± 1.44	4.38 ± 1.75	2.69 ± 1.49	−1.69 ± 1.03	0.002 *	**1.35**
Quality of life								
Physical functioning	89.23 ± 10.45	95.69 ± 11.62	6.46 ± 3.57	89.61 ± 6.81	92.53 ± 7.77	2.92 ± 2.13	0.006 *	**1.20**
Bodily pain	77.53 ± 5.34	83 ± 4.35	5.46 ± 4.53	79.84 ± 3.26	84.84 ± 2.91	5 ± 3.65	0.778	0.11
Physical role limitations	76 ± 7.77	84 ± 8.61	8.07 ± 7.27	81.07 ± 3.45	87.07 ± 4.29	6 ± 3.89	0.373	0.36
Emotional role limitations	85.3 ± 5.34	91.46 ± 7.41	6.15 ± 4.96	84.38 ± 3.92	88.92 ± 4.31	4.53 ± 2.72	0.314	**0.41**
Mental health	70.84 ± 5.33	77.23 ± 4.18	6.38 ± 3.9	67.76 ± 3.65	72.3 ± 3.83	4.53 ± 3.04	0.191	**0.53**
Social functioning	84.23 ± 4.02	89.76 ± 4.83	5.53 ± 2.69	89.53 ± 4.4	94 ± 5.61	4.46 ± 1.94	0.254	**0.46**
Energy/vitality	61.23 ± 4.22	65.76 ± 4	4.53 ± 2.14	66.61 ± 4.21	69.23 ± 4.24	2.61 ± 1.44	0.013 *	**1.05**
General health perceptions	71.46 ± 6.05	76.46 ± 4.17	5 ± 3.71	70.3 ± 3.52	74.07 ± 3.42	3.76 ± 2.74	0.347	0.38
SF-36 Total	615.84 ± 28.41	663.46 ± 29.37	47.61 ± 13.41	629.15 ± 13.42	663 ± 13.87	33.84 ± 10.49	0.456	**1.14**

* *p* < 0.05. † inter-group comparison. Data are shown as mean ± SD (Independent samples *t*-test; significance level *p* < 0.05).

## Data Availability

The raw data supporting the conclusions of this article will be made available by the authors on request.

## Abbreviations

The following abbreviations are used in this manuscript:

KT	Kinesiology Taping
PT	Placebo Taping
BMI	Body Mass Index
VAS	Visual Analog Scale
TSK	Tampa Scale of Kinesiophobia
ROM	Range of Motion
SF-36	Short Form of 36 Health Survey
